# Outcomes after minimally invasive lumbar decompression: a biomechanical comparison of unilateral and bilateral laminotomies

**DOI:** 10.1186/s12891-015-0659-2

**Published:** 2015-08-19

**Authors:** Yi-Hung Ho, Yuan-Kun Tu, Chih-Kun Hsiao, Chih-Han Chang

**Affiliations:** Department of Biomedical Engineering, National Cheng Kung University, No.1, University Road, Tainan, 701 Taiwan; Department of Orthopedics, E-DA Hospital, No.1, Yida Road, Jiaosu Village, Yanchao District Kaohsiung, 824 Taiwan; Department of Medical Research, E-DA Hospital, No.1, Yida Road, Jiaosu Village, Yanchao District Kaohsiung, 824 Taiwan

## Abstract

**Background:**

The unilateral approach for bilateral decompression was developed as an alternative to laminectomy. Unilateral laminotomy has been rated technically considerably more demanding and associated with more perioperative complications than bilateral laminotomy. Several studies have indicated that bilateral laminotomy are associated with a substantial benefit in most outcome parameters and thus constituted a promising treatment alternative. However, no complete kinematic data and relative biomechanical analysis for evaluating spinal instability treated with unilateral and bilateral laminotomy are available. Therefore, the purpose of this study was to compare the stability of various decompression methods.

**Methods:**

Ten porcine lumbar spines were biomechanically evaluated regarding their strain and range of motion, and the results were compared following unilateral or bilateral laminotomies and laminectomy. The experimental protocol included flexion and extension in the following procedures: intact, unilateral or bilateral laminotomies (L2–L5), and full laminectomy (L2–L5). The spinal segment kinematics was captured using a motion tracking system, and the strain was measured using a strain gauge.

**Results:**

No significant differences were observed during flexion and extension between the unilateral and bilateral laminotomies, whereas laminectomy yielded statistically significant findings. Regarding strain, significant differences were observed between the laminectomy and other groups. These results suggest that laminotomy entails higher spinal stability than laminectomy, with no significant differences between bilateral and unilateral laminotomies.

**Conclusions:**

The laminectomy group exhibited more instability, including the index of the range of motion and strain. However, bilateral laminotomy seems to have led to stability similar to that of unilateral laminotomy according to our short-term follow-up. In addition, performing bilateral laminotomies is easier for surgeons than adopting a unilateral approach for bilateral decompression. The results provide recommendations for surgeons regarding final decision making. Future studies conducting long-term evaluation are required.

## Background

The main purpose of decompression surgery is to alleviate the symptoms of nerve root compression and prevent further compression of the nerve roots [1]. In recent years, minimally invasive treatment for decompression has become widely practiced for achieving effective operation and maintaining the stability of the spine.

The advantages of unilateral approach for bilateral decompression involve minimising damage to the bone structure and maintaining original spinal biomechanical stability, which facilitates the prevention of postoperative spinal deformation and displacement. Both unilateral and bilateral laminotomies can effectively relieve severe nerve root compression symptoms. However, unilateral laminotomy has been rated technically considerably more demanding and associated with more perioperative complications than bilateral laminotomy; by contrast, bilateral laminotomy is associated with a substantial benefit in most outcome parameters and thus constitutes a promising treatment alternative [2]. According to Oertel et al., the incidence of complications related to unilateral laminotomy was 9.8 %. These complications included deep wound infection, disturbance of wound healing, and incidental durotomy [3].

In recent years, numerous studies have examined postoperative spinal instability, range of motion (ROM), and stiffness [4, 5]. Lee et al. assessed the differences in motion patterns after bilateral laminotomy and after laminectomy, concluding that full laminectomies increased in the L2–L5 range of motion by an average of 32.0 %, whereas bilateral laminotomy resulted in an average increase of 14.3 % [6]. Bresnahan et al. obtained similar results, demonstrating that removing posterior elements for treating stenosis at L3–L4 and L4–L5 resulted in increased flexion-extension and axial rotation [7]. However, regarding ROM, other studies have yielded different findings. Dahdaleh et al. demonstrated that laminectomy was not associated with significant increases in motion compared with intact spines with any load or in any direction [8, 9]. In summary, most studies have focused on investigating the differences between laminectomy and laminotomy; however, no complete kinematic data and relative biomechanical analysis for evaluating spinal instability treated by adopting a unilateral approach and bilateral laminotomies have been presented.

When decompression of the lumbar spinal canal is treated, segmental stability might be affected. Determining precisely which anatomical segments can be resected without hindering stability is crucial. The definition of instability in most studies indicates a sagittal translation greater than 3–4 mm or an angle change greater than 10–15° between adjacent vertebral bodies [10]. Delank et al. proposed that in adjacent segments, a slightly higher range of mobility is observed in rigid stabilisation than in dynamic stabilisation [11].

In clinical practice, surgeons often reserve the dorsal midline structure and perform laminotomy to decompress the nerves associated with unilateral or bilateral approaches. In recent years, the concept of minimally invasive surgery has become widely discussed and unilateral approaches for bilateral decompression have been increasingly adopted. However, unilateral laminotomy has led to more perioperative complications than bilateral laminotomy. To investigate the outcomes of these treatments, we constructed a motion simulator platform to facilitate experimentation using a porcine model. We developed an experimental platform to investigate the biomechanical mechanism of spinal decompression and clarify the outcomes of treating spinal canal stenosis. We hypothesized that the unilateral approach for bilateral decompression has a more positive effect than laminectomy but does not occur after bilateral laminotomy.

## Methods

### Uniquely designed motion simulator

**Fig. 1:**
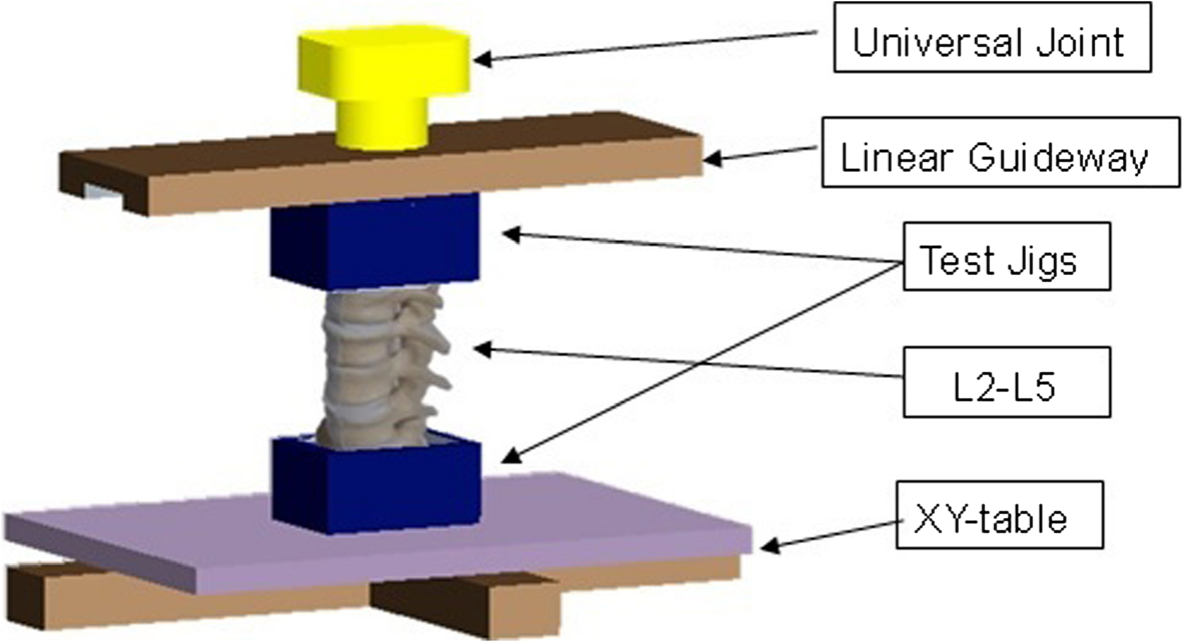
The unique-designed motion simulator

To ensure accurate ROM measurements, a motion simulator was designed for the experiment (Fig. 1). The main structural components are described as follows:

1. Material testing machines (MTSs): To provide a loading for our experiment. The displacement for each load was recorded (Instron 5848, USA).
2. Universal joint: To assist providing an axial loading.
3. Linear guideway: This was adopted to provide pure moment on the topside of the specimens.
4. XY-table: This table enabled the specimen subjected to the measurement system to have free movement so that they could be directly driven using the mechanical testing machine.
5. Sample jig: The bottom component fixed the specimen, and the top part to enable applying force.

The specimens in this study were tested in flexion (8 Nm) and extension (6 Nm) of pure moment. The MTSs applied 400 N at two centimetres from the top centre of the specimen to create pure flexion moment and 300 N for extension moment. A total of 400 N compressive-follower preload was applied during the flexion and extension tests [6].

### Specimen preparation

**Fig. 2:**
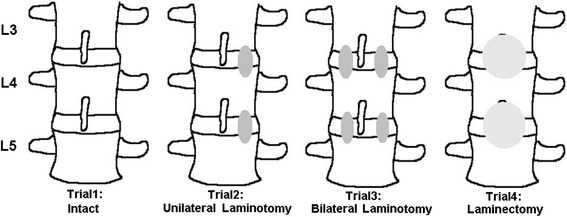
The experiment flow and explanation for four trials

For this study, we bought 10 porcine lumbar spines (L2–L5) at a traditional market, and ensured that motion redistribution over the entire mobile region was possible in all the specimens. To prepare each specimen for testing, the residual muscular tissues were removed, and all ligament and disc tissue was left intact. The testing specimen consisted of four vertebral bodies (L2–L5) with the intervening disc, posterior structures, and ligaments. In the intact group, all posterior components were preserved. Fenestration was performed on a single side at L3 and L4. In the unilateral approach for bilateral decompression group (unilateral laminotomy), the inferior margins of L3 and L4 lamina and the superior margins of L4 and L5 lamina were detached through using burr, and the ligamentum flavum was undercut. The L3, L4, and L5 supraspinous ligaments were preserved in all specimens. In the bilateral laminotomy group, fenestration was performed on both sides. Finally, the lamina and spinous processes of the lower L3 and upper L4 were detached using a rongeur and Kerrison clamp. This was also conducted at the lower L4 and upper L5. The supraspinous ligaments and ligamentum flavum of L3–L4 and L4–L5 were detached. All facet joints were preserved in all the specimens (Fig. 2).

### 3-D motion capture system

A three-dimensional motion capture system (Phoenix Techonlogies, Incorporated, PTI Canada) with a set of noncollinear lens markers was installed on each segment and applied to track the segmental motions. Three markers were placed on each vertebral body on L3, L4, and L5, with nine markers placed on the specimens. Kinematic data was recorded at a sampling rate of 10 Hz. The segmental ROMs between L3–L4 and L4–L5 were assessed.

### Test protocols

**Fig. 3:**
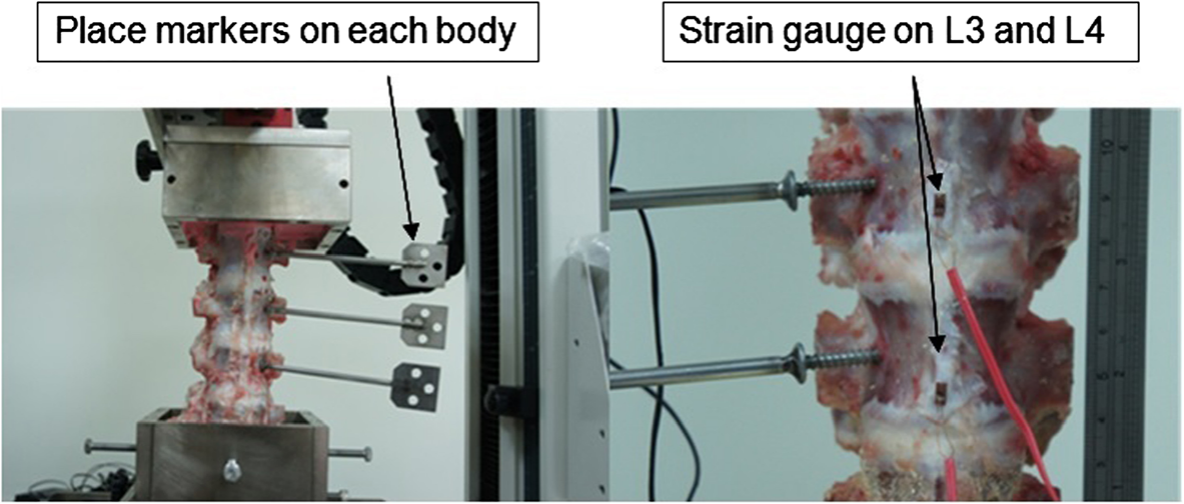
The picture of experiment setting

The experimental protocol included flexion and extension, which were tested under each of the following four conditions (trials). First, the spines were tested intact; subsequently, unilateral and bilateral laminotomy were performed, followed by laminectomy. The specimens were oriented in a neutral posture by rigidly mounting each end (L2 and L5) into the metal pots, with a test machine installed enabling the loads to be applied. Before the test, the strain gauge (KYOWA, KFG-1-120-C1-11, resistance: 120 Ω) was placed on the surface of the L3 and L4 bodies to measure the strain of each segment (Fig. 3). The signals were calibrated and amplified by connecting them to Vishay signal conditioners. National Instruments data translation board (National Instruments, Austin, TX, USA) and Labview 5.0 were used to record the signals.

### Data analysis and statistics

For the data analysis, a global coordinate system was defined according to the three markers that were set next to the specimens. These reference coordinates were relative throughout the entire experiment. The markers on the lumbar spine were defined as the local coordinate system. The relative motion between adjacent parts of the lumbar spine was obtained on the basis of computing the rotation matrix. In addition, the MTS and motion information were synchronised in the experiment, and the force and motion data were included. In the three-dimensional space, relative to the laboratory reference frame, a rotation matrix was used to represent object rotation. Distal segments relative to the reference frame, as well as proximal limb segments relative to the reference frame, were expressed using a rotation matrix. The movement of the spine was considered relative to the distal and proximal segments by referencing the natural anatomical position. The joint angle was computed using an Euler angle. The ROM at the surgical and inferior adjacent levels was evaluated using analysis of variance, and post hoc Tukey tests were performed to compare the differences (P ≤ 0.05) between the intact and surgical approaches.

## Results

The simulated motion involved combining the ROM and strain, to estimate the instability of the lumbar spine. The simulation involved smoothly performing flexion and extension and could be reproduced.

### Range of motion

**Fig. 4:**
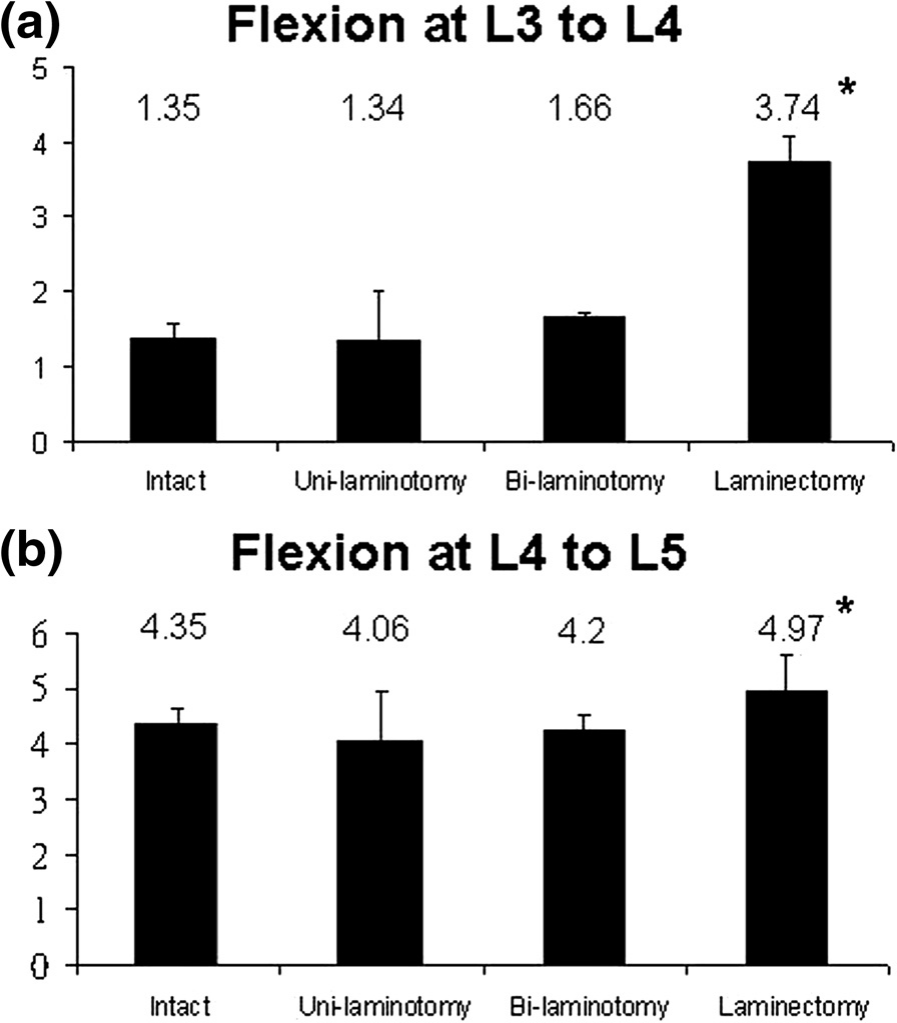
Degree of flexion at L3-L4 and L4-L5 in four groups

**Fig. 5:**
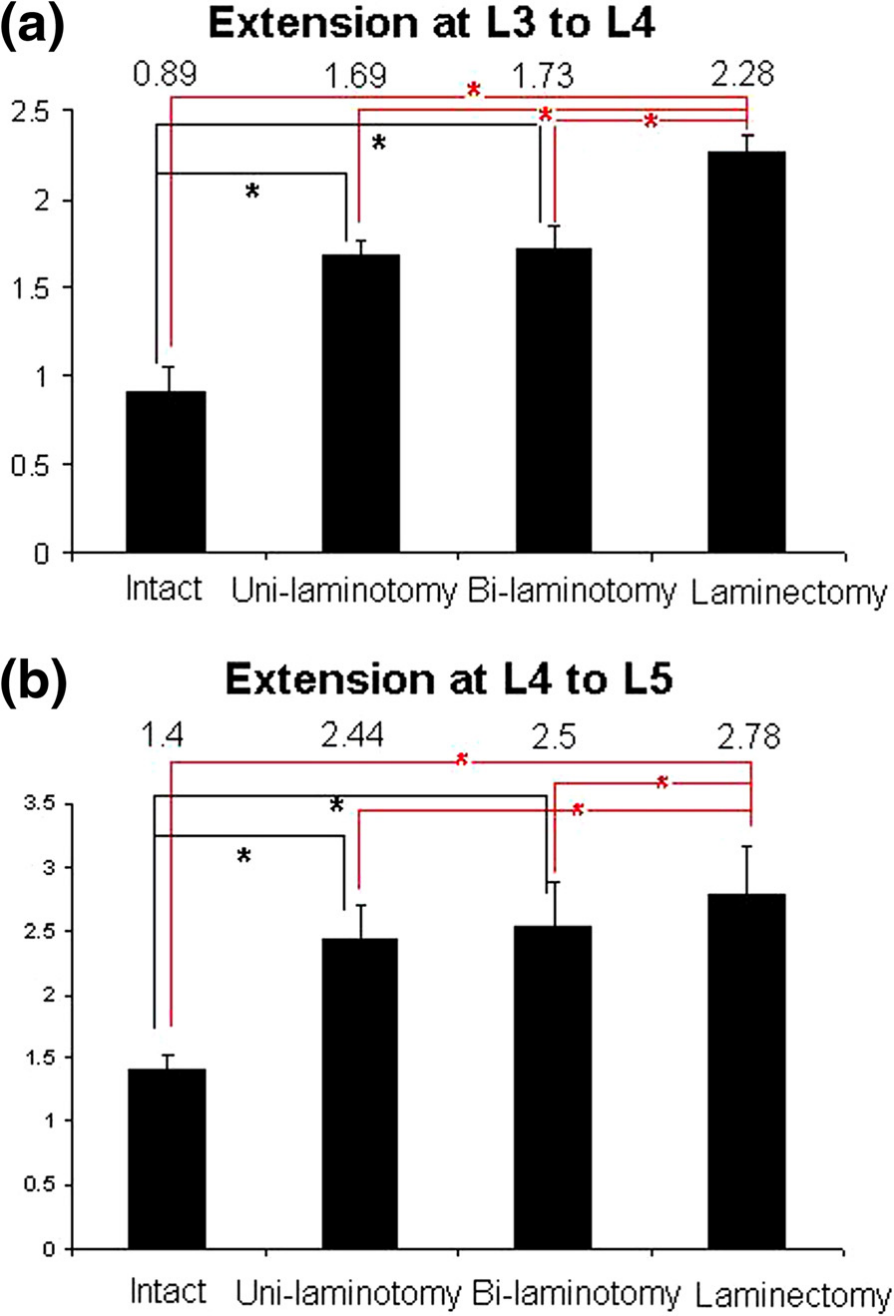
Degree of extension at L3-L4 and L4-L5 in four groups

The ROM was calculated on the basis on the locations of the markers tracked using the motion capture system. Flexion and extension data were collected from four trials. The L3–L4 and L4–L5 ROMs during flexion are illustrated in Fig. 4. In the flexion, the ROMs (±standard deviation) of L3–L4 were 1.35 ± 0.23, 1.34 ± 0.67, 1.66 ± 0.07, and 3.74 ± 0.35° of the intact, unilateral, bilateral laminotomies, and laminectomy, respectively. The ROMs (±standard deviation) of L4–L5 were 4.35 ± 0.29, 4.06 ± 0.87, 4.2 ± 0.32, and 4.97 ± 0.69°, respectively. However, significant differences were observed in the laminectomy in both the L3–L4 and L4–L5 groups (P < 0.05). No statistical significance was observed in the other groups (P > 0.05). Approximately 0.3 and 0.14° variation of L3–L4 and L4–L5 between the unilateral and bilateral laminotomy groups was observed; thus, no significant difference existed. Figure 5 shows the result of the extension. Significant differences were observed among all trials, except between the unilateral and bilateral laminotomy groups. Only 0.04 and 0.06° variation were observed between the unilateral and bilateral laminotomy groups. Therefore, we suggest that repeat tests are reasonable.

### Strain on the vertebral body

**Fig. 6:**
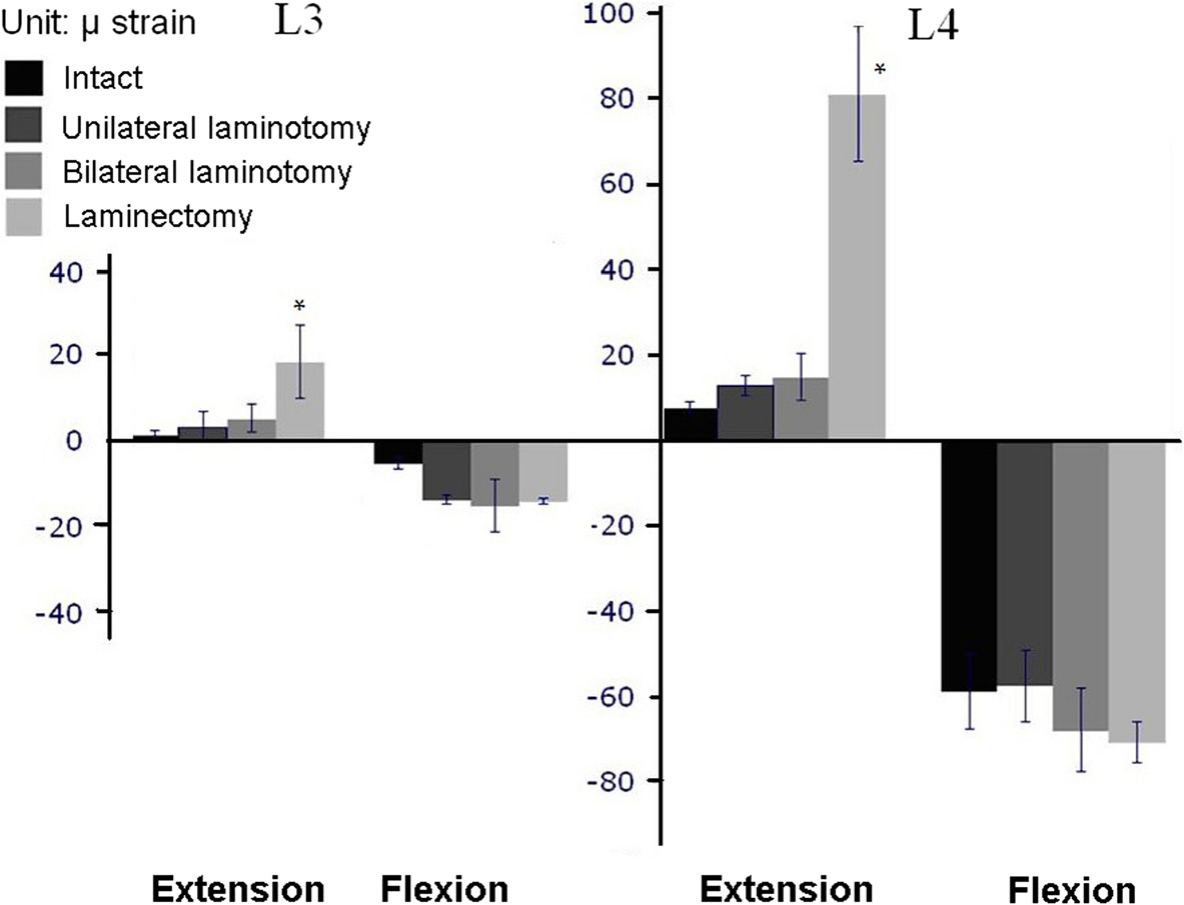
The average strain on L3 and L4 of four trials

The strain data were obtained using the acquisition system. Figure 6 illustrates the average strain on L3 and L4. The average strains (±standard deviation) during flexion of L4 were −58.64 ± 8.8, −57.23 ± 8.48, −67.68 ± 9.72, and −70.55 ± 4.88 micro strain of the intact, unilateral, bilateral laminotomies, and laminectomy, respectively. The same ROMs results were observed during flexion; however, a significant difference was observed in laminectomy in both L3 and L4. No statistical significance was observed in the other groups (P > 0.05). During extension, the strain results were the same as those that occurred during flexion. The average strains (±standard deviation) during extension of L4 were 7.86 ± 1.12, 12.9 ± 3.43, 15.06 ± 5.53, and 81.16 ± 15.8 micro strain of the intact, unilateral, bilateral laminotomy, and laminectomy, respectively.

## Discussion

The instability and mobility of the spine after stress relief exhibited relevant periods; However, how to simulate the motion of the spine has not been adequately defined [12]. In this study, we established a platform for simulating spinal movement and used the results to determine how to approach spinal stenosis. To understand the instability more clearly, the ROM and strain of the body should be further investigated. Thus, this goal was achieved in this study.

Regarding the unilateral and bilateral laminotomies, according to Thome’s study, unilateral laminotomy entailed more perioperative complications than did bilateral laminotomy. The complications included incidental durotomy, increased radicular deficit, and epidural hematoma [2]. The disadvantages of unilateral laminotomy were repoted by Oertel; a 11.8 % reoperation rate that was attributable to complication, restenosis, and spinal instability [3]. This research reexamined the necessity of the unilateral approach for bilateral decompression. From a biomechanical perspective, removing fewer elements is favorable. However, this was not apparent in flexion and extension. It may be linked that reserved the dorsal midline structure. This is one of our highly relevant discoveries. Inother words, we applied bilateral laminotomy to decrease complications but retained a similar stability using unilateral laminotomy.

Our results revealed a greater degree of flexion in the laminectomy group compared with other groups. These results have been also reported in Lee et al. and Bisschop et al., who compared laminectomy with an intact group in 2010 [6, 13]. Regarding flexion simulation, we recognised the contribution of the vertebral body and disc, particularly in the vertebral body under strain. No statistical significance was observed between laminotomy and the intact group during flexion; by contrast, opposing results were found during extension. If either laminotomy or laminectomy involved removing the posterior elements of the spine, it would affect the extension more than the flexion. During extension, the posterior element plays a crucial role in stoppingany motion, like a stopper. Unilateral and bilateral laminotomies causing a higher degree of extension than that observed in the intact spines may be attributable to the aforementioned reason.

Increased ROM after surgery generally indicates greater instability of the spine [14]. The findings of this study provide insight into preparing for spinal stenosis. If the affect on stress relief is the same, laminotomy may be a more favorable choice than laminectomy. Numerous factors influence surgical decision-making including the severity of stenosis, preoperative segmental mobility, and medical complications. The final decision may be made by surgeons according to each case, which Lee et al. also proposed [6].

The innovation of our study consists of using a strain gauge to measure the strain. The strain of L3 and L4 exhibited similar trends to that of ROM during flexion, which is consistent with our findings according to which the greatest contributor to ROM is the vetebral body. Various scholars have indicated that if the strain was concentrated on one vertebral body, this may indicate degeneration after treatment of spinal stenosis, as numerous studies have proposed [15].

In particular, we sought to determine the changes after applying loads and after having attempted to measure it previously. Compared with the unilateral approach and bilateral laminotomies during extension, we determined that the strain of L3–L4 was not any different, but both of them were more increased than intact. The reason may be that after removing the posterior elements, the disc loses the ability to support the entire spine and carry increased pressure. Additional obvious phenomena occurred in laminectomy. Previous research has concluded that more instances of degeneration occur after laminectomy [16].

Although efforts have been made to simulate clinical conditions, this study has numerous limitations. This experimental study involved using porcine lumbar spines. This is because that numerous studies [17–19] have evaluated the biomechanical behaviors of the spinal column by using porcine lumbar spines instead of the human spine. In addition, it has been established that certain regions of the porcine spine are qualitatively similar to the human spine. The vertebral body heightis highly similar in human and porcine vertebrae. Busscher concluded that the results between the human and porcine lumbar are comparable, which is evident through the superior classification of the intraclass and interclass correlations [18]. Futhermore, the specimens in our study were harvested from normal, mature porcine spines that lacked degeneration, instablity, and osteoporosis. An additional limitation was that this study was a short-term follow-up because of the motion simulator design limitation. Future studies should conduct long-term evaluations.

The purpose of the present study was to construct a platform for simulating the motion of the spine and comparing the stability of various decompression methods. During flexion, a greater ROM and body strainwere measured in the laminectomy group compared with the intact spine group. Furthermore, according to our results based on using ROM and strain on the vertebral body, no particular differences were observed between the unilateral and bilateral laminotomies. In clinical practice, the bilateral laminotomies are likely to reduce technical difficulties and prevent perioperative complications; this study proved this benefit through biomechanical analysis. Our findings provide a valuable reference for surgeons in the preparation for treatment. However, any final decision should always involve considering a patient’s individual clinical status. In future studies, we will test lateral bending and axial rotation by applying the same simulation concept.

## Conclusions

In conclusion, the present results reveal that no significant differences were observed during flexion between unilateral and bilateral laminotomies in short-term follow-up, whereas statistical significant findings were observed in laminectomy. Although unilateral laminotomy can be performed through adopting minimally invasive approaches, it entails an increased risk of perioperative complications. From a biomechanical point of view, bilateral laminotomies seem to exhibit a similar stability as unilateral laminotomy in short-term follow-up. Future studies should conduct long-term evaluations. However, any final decision should always involve considering the patient’s individual clinical status.

## Abbreviations

ROM: Range of motion
MTSs: Material testing machines
